# Systemic antihyperalgesic effect of a novel conotoxin from *Californiconus californicus* in an inflammatory pain model

**DOI:** 10.3389/fpain.2024.1500789

**Published:** 2025-01-24

**Authors:** Joaquín López-Carrillo, Johanna Bernáldez-Sarabia, Tushar J. Pawar, Samanta Jiménez, Salvador Dueñas, Andrea Figueroa-Montiel, José L. Olivares-Romero, Vinicio Granados-Soto, Alexei F. Licea-Navarro, Nadia L. Caram-Salas

**Affiliations:** ^1^ Departamento de Innovación Biomédica, CICESE, Ensenada, Mexico; ^2^ Red de Estudios Moleculares Avanzados, Instituto de Ecología (INECOL), Xalapa, Mexico; ^3^ Neurobiology of Pain Laboratory, Departamento de Farmacobiología, Cinvestav, South Campus, Mexico City, Mexico; ^4^ Dirección de Impulso a la Innovación y el Desarrollo (DIID), CICESE, Ensenada, Mexico; ^5^ CONAHCYT. Av. Insurgentes Sur 1582, Col. Crédito Constructor, Deleg Benito Juárez, Mexico City, Mexico

**Keywords:** conotoxin systemic administration, *Californiconus californicus*, chronic pain treatments, analgesia, inflammatory pain

## Abstract

**Introduction:**

This study explores the analgesic potential of the novel conotoxin O1_cal6.4b, derived from *Californiconus californicus*, as a candidate for pain management in a model of inflammatory pain.

**Methods:**

O1_cal6.4b was systemically administered to Wistar rats, and its effects on thermal hyperalgesia and motor coordination were evaluated. Comparative analyses were conducted against O1_cal6.4d, *ω*-MVIIA, and standard analgesics (morphine, dexamethasone, and diclofenac). Structural differences between O1_cal6.4b and O1_cal6.4d were examined using in silico modeling and molecular dynamics simulations.

**Results:**

Systemic administration of O1_cal6.4b significantly reduced thermal hyperalgesia in a dose-dependent manner without impairing motor coordination. The analgesic effect of O1_cal6.4b was superior to that of O1_cal6.4d, *ω*-MVIIA, and standard analgesics. Structural analyses revealed notable differences between O1_cal6.4b and O1_cal6.4d, suggesting unique functional properties.

**Discussion:**

The findings indicate that O1_cal6.4b exhibits a promising analgesic profile with advantages over traditional opioid-based therapies. These results underscore the molecular diversity of conotoxins and highlight their potential as innovative analgesic treatments. Further research is needed to elucidate the mechanism of action of this novel conotoxin.

## 1 Introduction

Pain is a complex and multifaceted phenomenon encompassing sensory and emotional experiences associated with actual or potential tissue damage. It serves a vital protective evolutionary function in response to a chemical, thermal, or mechanical stimulus to avoid potentially harmful situations (1, 2). It results from activating the nociceptors at the site of tissue damage. While acute pain is adaptive, chronic pain is a pathological condition that persists for more than six months, often continuing after the resolution of the initial injury or due to nervous system damage (neuropathic pain) (2, 3). Chronic pain induces allodynia (pain from normally non-painful stimuli) and hyperalgesia (increased sensitivity to painful stimuli), leading to significant somatic and psychological burdens, such as tension, anxiety, and depression, which severely diminish patients’ quality of life (4, 5).

Current treatments for chronic pain include opioids, non-steroidal anti-inflammatory drugs (NSAIDs), selective COX-2 inhibitors, antidepressants, anticonvulsants, and local anesthetics (6). These drugs often provide limited relief and are associated with significant side effects, such as gastrointestinal damage, constipation, tolerance, and dependence (7, 8). Consequently, there is an urgent need for novel analgesic agents that are effective, but with a more favorable side-effect profile. Research on the potential pharmacological use of venom-based drugs is ongoing (9, 10). Most drugs used in Western medicine have a natural origin. Animal venoms in recent years have emerged as an essential source of potential new drugs (11). For instance, in the field of pain therapeutics, toxins from snakes, spiders, or marine snails are being investigated (9, 11, 12). Marine cone snails are found mainly in tropical waters of the western Indo-Pacific Ocean, with some species that have adapted to more temperate waters around South Africa, the Mediterranean, and the southern Californian coast (13). At present, more than 800 species of cone snails have been identified and distributed in 57 distinct clades (or subgenera) (14, 15). Each species has a distinct, tailored, and selective venom cocktail of more than 1,000 unique peptides called conotoxins or conopeptides, and small molecules of non-peptidic nature (15, 16). Depending on their prey, cone snails can be classified as piscivorous (fish-hunting), molluscivorous (mollusks-hunting), and vermivorous (polychaetes-hunting) (17, 18). However, in some cases cone snails have more than a single type of prey, like *Californiconus californicus*, previously named *Conus californicus*. According to Biggs et al. (19), this species can feed on fish, mollusks, worms, and crustaceans.

Conotoxins, for example *ω*-MVIIA (ziconotide, Prialt®), have been identified as compounds that can induce analgesia in chronic pain by modulating N-type voltage-gated calcium channels. *ω*-MVIIA (ziconotide) and *ω*-GVIA are restricted to the infusion into the spinal cord due to some difficulties such as poor bioavailability, susceptibility to cleavage by proteases, and unwanted side effects (20, 21). Other *ω*-conotoxins are effective only after intracerebroventricular administration (22). The disadvantages of ziconotide by the intrathecal route include serious side effects (dizziness, nausea, confusion, and nystagmus), careful dose titration, narrow therapeutic window, and complications related to the intrathecal pump (21). In addition, ziconotide may induce cognitive impairment (mental slowing, impaired memory and speech, confusion, psychosis, changes in consciousness) (23). Ziconotide is used in patients with chronic refractory pain (cancer or HIV infection). Thus, alternative administration routes would represent a significant improvement in the field of pain management. In this context, leconotide (*ω*-CVID), an analog of *ω*-MVIIA, can be administered intravenously, while GVIA, another *ω* conotoxin isolated from *Conus geographus*, can be administered subcutaneously (20, 24). Unfortunately, both demonstrate limited antihyperalgesic effects. Thus, development of a conotoxin with greater efficacy than leconotide or other conotoxins and that can be administered intraperitoneally would be a great advance in pain therapeutics.

*Californiconus californicus* is a cone snail species distributed from San Francisco, CA, USA, to Baja California, Mexico (19). Research about *C. californicus* venom has exhibited the existence of numerous conotoxins with therapeutic potential. For instance, Bernáldez-Sarabia et al. (25) identified O1_cal29b, a conotoxin capable of inhibiting the growth of *Mycobacterium tuberculosis*. In addition, Oroz-Parra et al. (26) reported the cytotoxic impact of two synthetic peptides, Cal14.1a and Cal14.1b, on the lung cancer cell line H1299. More recently, Lugo-Fabres et al. (27) documented that the synthetic conotoxin s-cal14.2b modulated insulin secretion *in vitro* and reduced blood glucose levels *in vivo*. These research findings highlight the therapeutic potential of conotoxins from *C. californicus*. Of note, O1_cal6.4b, derived from *C. californicus* as well as ziconotide are members of the same superfamily (O1) and share the pattern of cystine knot structural motif (C–C–CC–C–C) (28–31). This makes O1_cal6.4b a potential candidate for a drug with antinociceptive properties and prompted the present study. Regarding the intraperitoneal administration, we tested several conotoxins for this route with the idea of finding one with good efficacy and a low profile of adverse effects.

In this study, we investigated the antihyperalgesic effect of a synthetic conotoxin, O1_cal6.4b, derived from the venom of *C. californicus* in rats. In addition, we carried out the comparison with *ω*-MVIIA (ziconotide) and other standard analgesic drugs. Data indicate that systemic administration of conotoxin O1_cal6.4b, but not O1_cal6.4d, is highly effective in reducing thermal hyperalgesia in a rat model of inflammatory pain.

## 2 Materials and methods

### 2.1 Conotoxins

The conotoxin sequences O1_cal6.4b and O1_cal6.4d were derived from a transcriptome of the venom gland of *C. californicus*. Synthetic peptides were synthesized by Agentide Inc. (Westfield, NJ, USA). In addition, complete Freund's adjuvant (CFA; heat-killed *M. tuberculosis*), diclofenac, and dexamethasone were obtained from Sigma-Aldrich (St. Louis, MO, USA). Morphine was generously provided by the Secretaría de Salud (Mexico City, Mexico).

### 2.2 Transcriptome of the venom gland

#### 2.2.1 Total mRNA extraction

Specimens of *C. californicus* from the Pacific Ocean side of Ensenada, Baja California, Mexico, were used. Venom ducts were dissected from each cone snail under RNase-free conditions. Total RNA extraction was performed using the SV Total RNA Isolation System (Promega, Madison, WI, USA) following the manufacturer's instructions.

#### 2.2.2 RNA-Seq library and transcriptome assembly of venom ducts

A DNA library was constructed using the TruSeq Stranded mRNA Sample Preparation Kit (Illumina, San Diego, CA, USA), following the supplier's and the Institute of Biotechnology in Cuernavaca, Mexico, protocols using an Illumina Genome Analyzer IIx and a 72 bp paired-end sequencing scheme over cDNA fragments ranging in size from 200 to 400 bp. The quality of the raw reads was assessed using the FastQC program (Illumina, San Diego, CA, USA). In the absence of a reference genome for the cone snail analyzed, short reads were assembled *de novo* into contigs using Trinity software v. 2.0.3 (32), following the standard protocol provided by the software developers (33). The quality of the assembly, basic statistics for the number of genes and isoforms, as well as contiguity were obtained by running the TrinityStats.pl script. Bowtie2 (34) and the integrative tool Genomic Viewer were used to display the compiled contiguity (35).

#### 2.2.3 Bioinformatic analysis of conotoxin identification

After assembly, open reading frames longer than 50 amino acids were generated using the Transdecoder utility included in Trinity. Redundant sequences were removed, retaining only those that began with methionine and a signal peptide after translating the peptide sequences. The signal peptide sequences were determined using the signalP4.1 server tool.

To detect new conotoxins from the filtered sequences, a Hidden Markov Model (HMM) profile was generated. This process was carried out using a similar method described in Robinson et al. (36) and Peng et al. (37). It involved aligning the sequences of each conopeptide superfamily, which was performed using MAFFT 7.0 (38). Subsequently, the multiple alignments of the sequences were utilized as input to create the pHMM profiles using HMMER 3.0. Finally, these profiles were employed to identify the conotoxins represented in the transcriptome.

### 2.3 *In silico* modeling

#### 2.3.1 Homology modeling

MODELLER v.9.20 software (39) was used for the 3D structure prediction of two conotoxins (cal6.4b and cal6.4d). This was performed through “Advanced Modeling,” which is based on multiple templates. To identify potential consensus templates for modeling, a protein BLAST was conducted. Conotoxins were modeled based on three PDB files of the conotoxin MVIIA protein scaffold (PDB ID, 1FEO, and 1DW4 1OMG).

#### 2.3.2 Molecular dynamics refinement

Structures were refined using the simulated annealing strategy of the NAMD software. The molecular graphics software Visual Molecular Dynamics (VMD) (40) was used to prepare the structures for the application of a force field using the Chemistry at Harvard Macromolecular Mechanics (CHARMM36) tool before molecular dynamics calculations. VMD was also used for post-analysis of the root mean square deviation (RMSD) and root mean square fluctuation (RMSF) plots. The simulations were conducted in a water box as the solvent in all cases, employing periodic boundary conditions and assuming an NPT ensemble with a constant number of particles (N) and constant isobaric (P) and isothermal (T) conditions. The pressure was set at 1 atm and the temperature at 300 K. These periodic boundary conditions were iteratively coupled with annealing and relaxation steps. After the annealing and cooling, each conotoxin was subjected to molecular dynamics analysis at 300 K and 1 atm for 100 ns. The analysis of atomic trajectory coordinates and energies was written to disc every 10 ps. After simulating the annealing and molecular dynamics calculations, the different conformational structures were grouped based on their overall energy stability throughout the simulation. To find the most thermodynamically stable protein conformation, the structure with the longest lifetime was selected. PyMOL Molecular Graphics System v2.2.2 was used to visualize the refined conotoxins.

### 2.4 Experimental procedures

#### 2.4.1 Animals

Female and male Wistar rats weighing between 120 and 140 g were obtained from the CICESÉs animal facility for this study. These rats were accommodated in acrylic cages, with six rats per cage (44 cm wide × 33 cm long × 20 cm high) with unrestricted access to water and food and their living environment was maintained at a controlled temperature of 22 ± 1°C following a light cycle of 12 h light and 12 h darkness (lights off at 10:00 h). The experimental procedures adhered to the guidelines outlined in the UK Animals (Scientific Procedures) Act, 1986 and associated guidelines, the Council Directive of the European Communities of 24 November 1986 (86/609/EEC) or the National Institutes of Health guide for the care and use of laboratory animals (NIH Publications No. 8023, revised 1978) and the Mexican regulation NOM-062-ZOO-1999. Furthermore, these procedures received approval from the Institutional Committee on the Care and Use of Laboratory Animals at CICESE, Baja California, Mexico (Protocol number 2023–02). At the end of each experiment, animals were euthanized using a CO_2_ chamber according to the AVMA Guidelines for the Euthanasia of Animals.

#### 2.4.2 Model of inflammatory hyperalgesia

Inflammatory hyperalgesia was induced by injecting a small volume (100 µl) of CFA suspended in oil: saline emulsion (1:1) into the ipsilateral (right) paw. Paw withdrawal latency in response to the application of a radiant stimulus to the plantar surface of the right and left paws was measured using the Plantar Analgesia Meter device for paw stimulation (IITC Life Science, Woodland Hills, CA, USA). To avoid a heat sink, the temperature of the glass plate was maintained at 29°C; the intensity of the radiant heat light source was adjusted to 20%, resulting in an approximate temperature of 45°C on the underside of the glass. The time taken for the animal to respond by licking or flicking its paw was interpreted as a positive response (paw withdrawal latency). A cut-off time (20 s) was set to automatically turn off the heat source to avoid tissue damage (41).

Two days before the CFA injection, the light-intensity lamp was adjusted to elicit baseline paw withdrawal latencies between 17 and 20 s in both paws for each animal. The paw withdrawal latency was performed by measuring each 30 min to complete 5 h, then each 24 h until 96 h after right paw CFA injection to obtain the development of thermal hyperalgesia. Two days after the CFA injection and before the administration of the drugs, the baseline was measured again in both paws to confirm the development of thermal hyperalgesia. The use of 2 days was based on previous publications (41, 42). The contralateral (non-inflamed) paw was always used as control. Animals with latency times more than 6 s in the inflamed paw and less than 17 s in the normal paw were excluded from further experiment. On the day of the experiment, the animals were acclimatized in the analgesia meter equipment for more than 30 min.

The following treatments were tested: O1_cal6.4b (0.001–1 mg/kg, testing conotoxin), O1_cal6.4d (1 mg/kg, conotoxin control), *ω*-MVIIA (0.5 mg/kg, conotoxin control), saline (1 ml/kg, vehicle), dexamethasone (1–2 mg/kg, positive control), morphine (1–10 mg/kg, positive control), and diclofenac (1–10 mg/kg, positive control). Doses of in-house conotoxins (1 mg/kg) used in this study were selected from pilot experiments in our conditions. Systemic dose of *ω*-MVIIA (0.5 mg/kg) was selected from the literature considering that the highest no-side-effect dose of this drug is 0.02 mg/kg (20). However, since at this dose it did not induce any effect, we preliminarily tested a dose enough to induce a mild antihyperalgesic effect, which amounted to 0.5 mg/kg. Doses of dexamethasone (2 mg/kg), morphine (10 mg/kg), and diclofenac (10 mg/kg) were selected from the literature (43–45). All drugs were administered intraperitoneally. The withdrawal latency of the ipsilateral paw (inflamed) and contralateral (control) was measured at 30-min intervals over 8 h for the antihyperalgesic activity of the drugs.

#### 2.4.3 Motor coordination test

Since *ω*-MVIIA (ziconotide) may affect motor coordination (46), the effect of the greatest tested dose of O1_cal6.4b and *ω*-MVIIA were assessed. Both groups were tested for possible side effects using the Rota-rod system assay according to a previous study (47). Animals were trained for 3 days by walking them each day on an accelerating Rota-rod apparatus (Panlab 8500, Cornellá BCN, Spain). Rats were placed on a cylinder (7 cm in diameter) rotating at a speed of 5 rpm for 10 min, then at a speed of 10 rpm for 5 min after being trained to walk on the cylinder for three consecutive sessions (47). On the fourth day, the rats received vehicle (1 ml/kg), O1_cal6.4b (1 mg/kg) or *ω*-MVIIA (0.5 mg/kg) at time 0, and the number of falls during 5 min was counted after 1 h post-drug administration. This time was chosen based on the time to reach the maximal antihyperalgesic effect of O1_cal6.4b.

#### 2.4.4 Statistical analysis

All results are presented as mean ± SEM of five to six animals per group. The area under the curve (AUC) was calculated using the trapezoidal method to derive the percentage of the maximum possible effect (%MPE), an expression of the antihyperalgesic effect, using the following formula:

$$\text{\%}MPE=\left(\frac{AUC_{molecule}-AUC_{inflamed\;paw}}{AUC_{normal\;paw}-AUC_{inflamed\;paw}}\right)\times 100$$

where $AUC_{inflamed\;paw}$ and $AUC_{normal\;paw}$ are the values obtained from the vehicle (saline)-injected group.

Thermal hyperalgesia between male and female rats were analyzed by an unpaired Student's *t*-test. In the study of the antihyperalgesic activity of O1_cal6.4b, one-way ANOVA followed by the Dunnett test was used to compare differences between treatments and saline group. Differences were considered statistically significant when *P* ≤ 0.05. Finally, to determine the median effective dose (ED_50_), a linear regression was made with an interpolation of the sigmoidal curve. All statistical analyses were carried out on GraphPad Prism 9.3 (GraphPad Software Inc., San Diego, CA, USA).

## 3 Results

### 3.1 Transcriptomic analysis

Eighteen different superfamilies of conopeptides were identified from the *Californicus californicus* venom duct. Following transcriptomic analysis, homologous conotoxins cal6.4b and cal6.4d members of the O1 superfamily were found.

### 3.2 *In silico* modeling and molecular dynamics

The structural prediction of the conotoxins O1_cal6.4b and O1_cal6.4d was achieved through homology modeling and refinement via molecular dynamics. Structural differences between the homologous conotoxins were analyzed, considering sequence, structural alignments, RMSD, and RMSF of the models.

**Figure 1:**
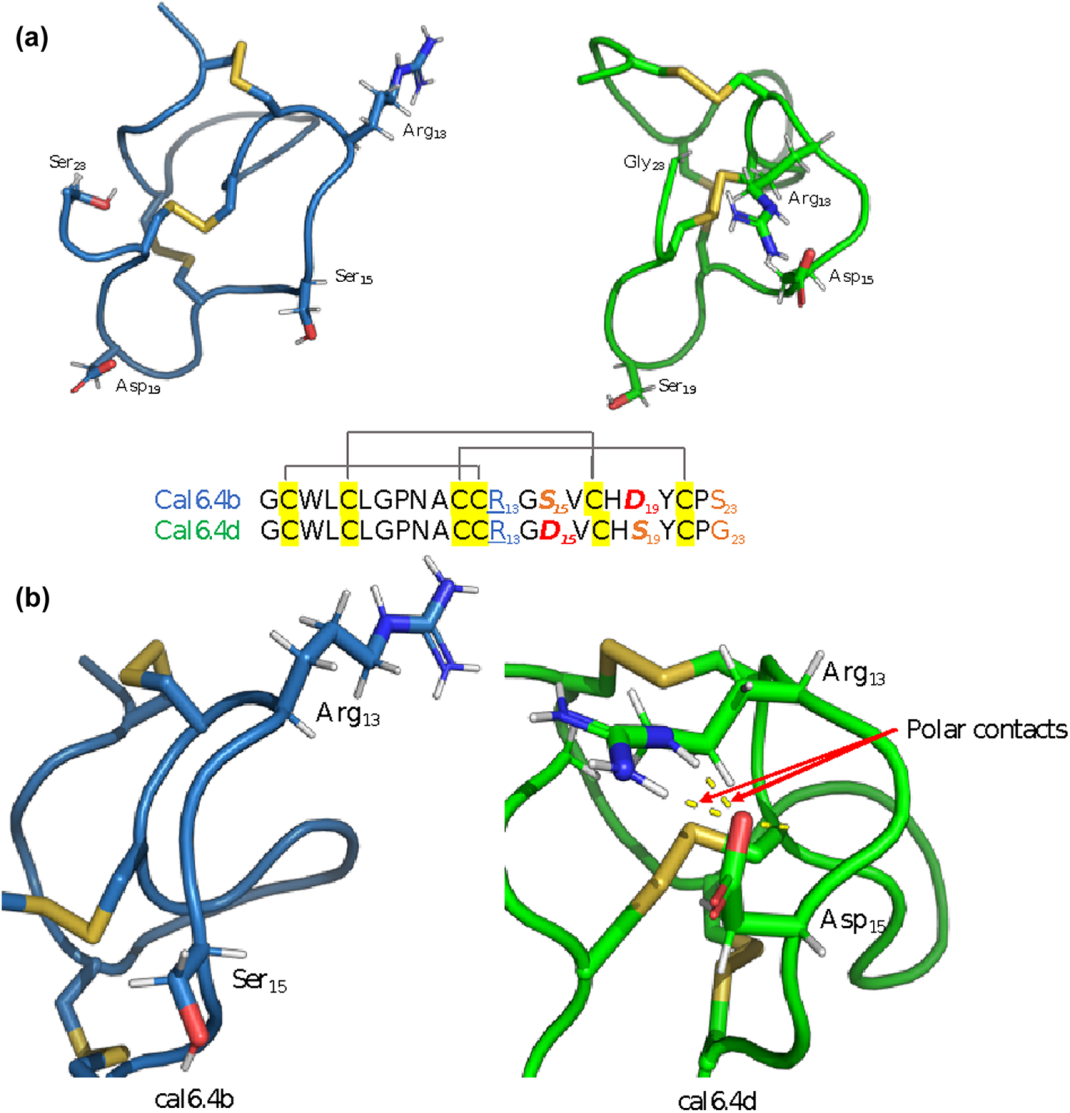
Refined models of O1_cal6.4b and O1_cal6.4d. (**a**) Colored in blue, the model of cal6.4b shows the different conformation against cal6.4d (green). (**b**) Zoomed-in view of the conotoxin structure shows the main difference between cal6.4b (free Arg13) and cal6.4d (joined Arg13). It indicated with red arrows the interaction between Arg13 and Asp15.

**Figure 2:**
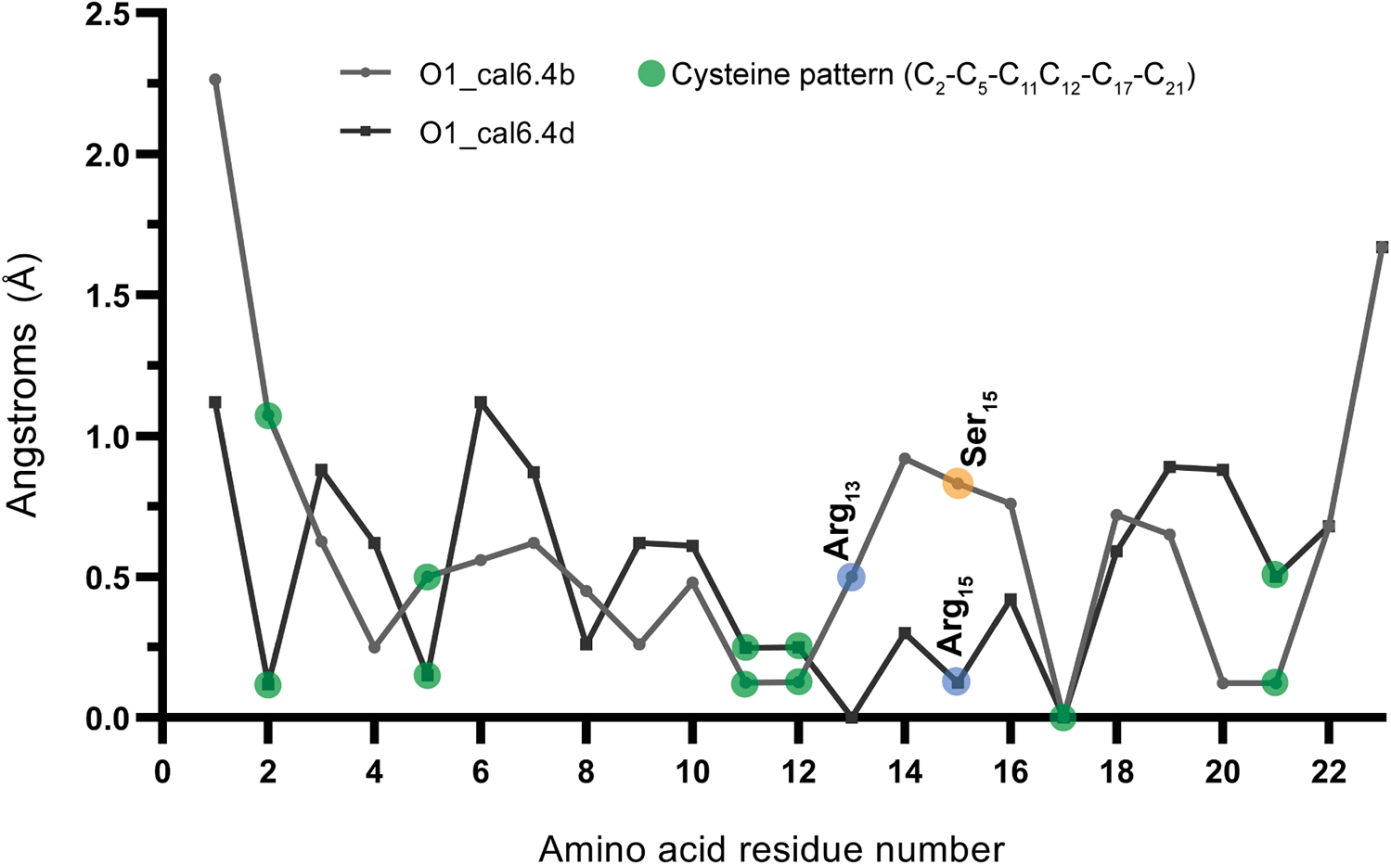
RMSF of models O1_cal6.4b and O1_cal6.4d. Indicating with blue and orange circles, the main difference between each conotoxin model (Ser15 and Asp15) also shows the fluctuation change of the Arg13 in the cal6.4b model compared with cal6.4d.

While both conotoxins exhibited a high degree of similarity (Figure 1a), a significant structural disparity arose due to a single amino acid substitution at position 15, with O1_cal6.4b featured serine and O1_cal6.4d featured aspartic acid. In O1_cal6.4b, serine's presence does not perturb the side chain of arginine 13, while in cal6.4d, aspartic acid attracts and forms polar contacts with arginine 13, leading to structural differences (Figure 1b). Regarding RMSD, cal6.4b exhibited fluctuating values ranging from 1.8 to 4.6 Å, achieving stability at five nanoseconds (Supplementary Figure S1a). By contrast, the model for cal6.4d displayed an RMSD range of 2.5–4.5 Å, attaining a stable conformation after nine nanoseconds (Supplementary Figure S1b). We further assessed the RMSF of both models. Despite the high sequence similarity, notable differences in side-chain mobility and amino acid fluctuation were observed. O1_cal6.4d exhibited a more rigid protein structure compared to cal6.4b. Figure 2 illustrates the distinctions in mobility within the region spanning from Arg13 to Val16 in both models. The rigidity in this segment of O1_cal6.4d can be attributed to the interaction absent in O1_cal6.4b, where Arg13 remains unbound as it does not engage with a negatively charged amino acid.

### 3.3 CFA-induced thermal hyperalgesia

**Figure 3:**
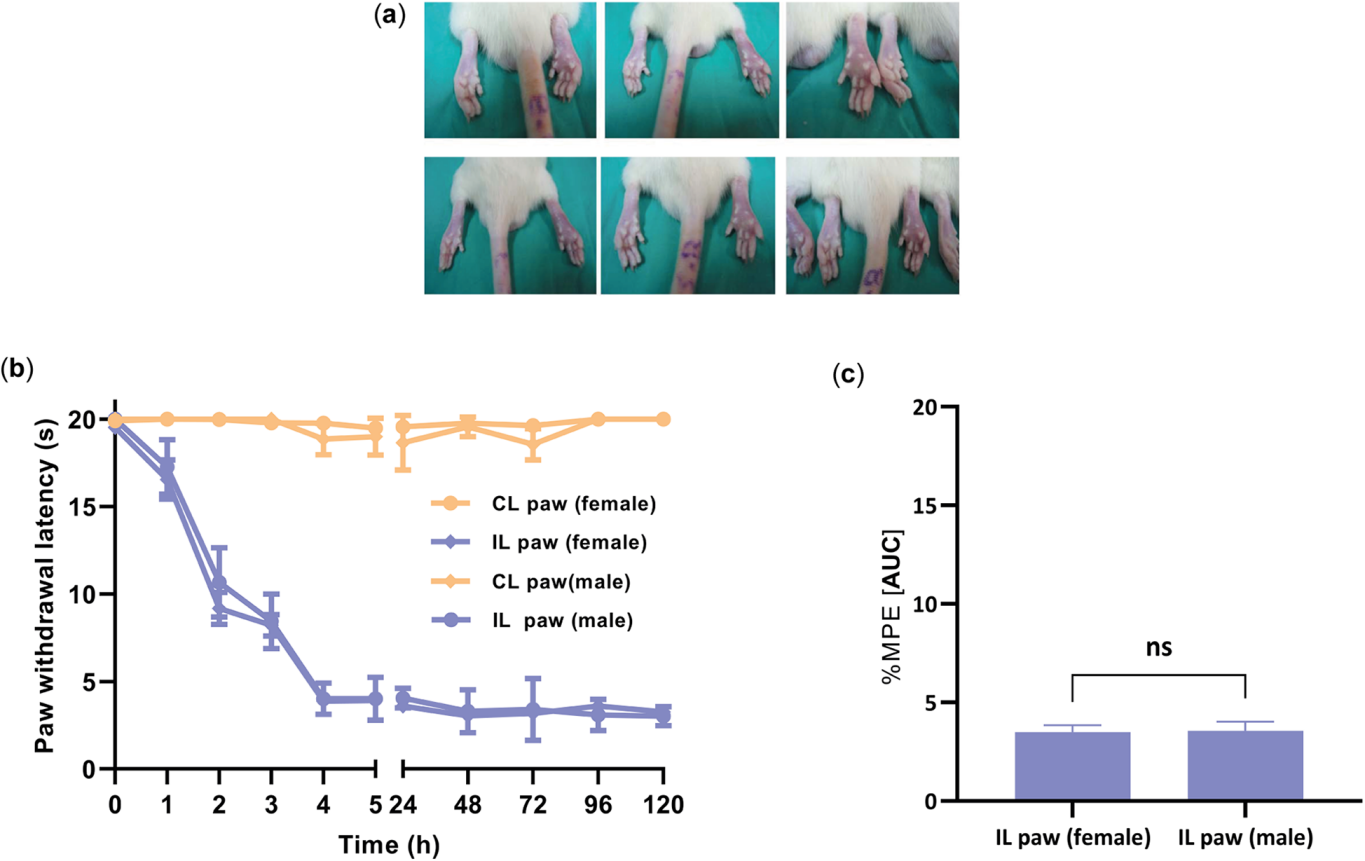
Development of hyperalgesia induced by the intraplantar injection of CFA into the ipsilateral paw (CFA paw) in female and male rats. (**a**) Edema changes of the ipsilateral paw after the CFA administration. (**b**) Time course of thermal hyperalgesia observed after the administration of CFA into the ipsilateral paw. The data are represented as the paw withdrawal latency in seconds in female and male groups. (**c**) Comparison of the withdrawal latency at 4 h between female and male groups after administration of CFA into the ipsilateral paw. All data are the mean ± SEM for five to six animals per group. *P* = 0.9264, as determined by the unpaired Student's *t*-test. IL, ipsilateral (paw treated with CFA); CL, contralateral paw (normal paw); ns, non-significant; s: seconds.

Administration of CFA (100 µl) to the right paw (ipsilateral) produced edema and thermal hyperalgesia in female and male rats (Figure 3), as evidenced by a decrease in the paw withdrawal threshold compared to the normal paw (contralateral) (Figure 3). The development of hyperalgesia was monitored every 30 min until 3 h, then every 24 h until 72 h. The mean paw withdrawal latency in the contralateral was 19.33 ± 0.42 s in the female group and 19.44 ± 0.23 s in the male group. Thermal hyperalgesia reached its peak around day 3 (72 h), with a withdrawal latency of 4.00 ± 0.19 s for the female group and 4.00 ± 0.22 s for the male group. No significant differences were observed in paw withdrawal latency between male and female rats at any time point after the establishment of hyperalgesia, particularly after the 4 h evaluation mark (*P* = 0.9264) (Figure 3c). Based on these results, we chose to use female rats to investigate the antihyperalgesic effect of O1_cal6.4b 2 days after the CFA paw injection.

### 3.4 Analgesic activity of O1_cal6.4b

**Figure 4:**
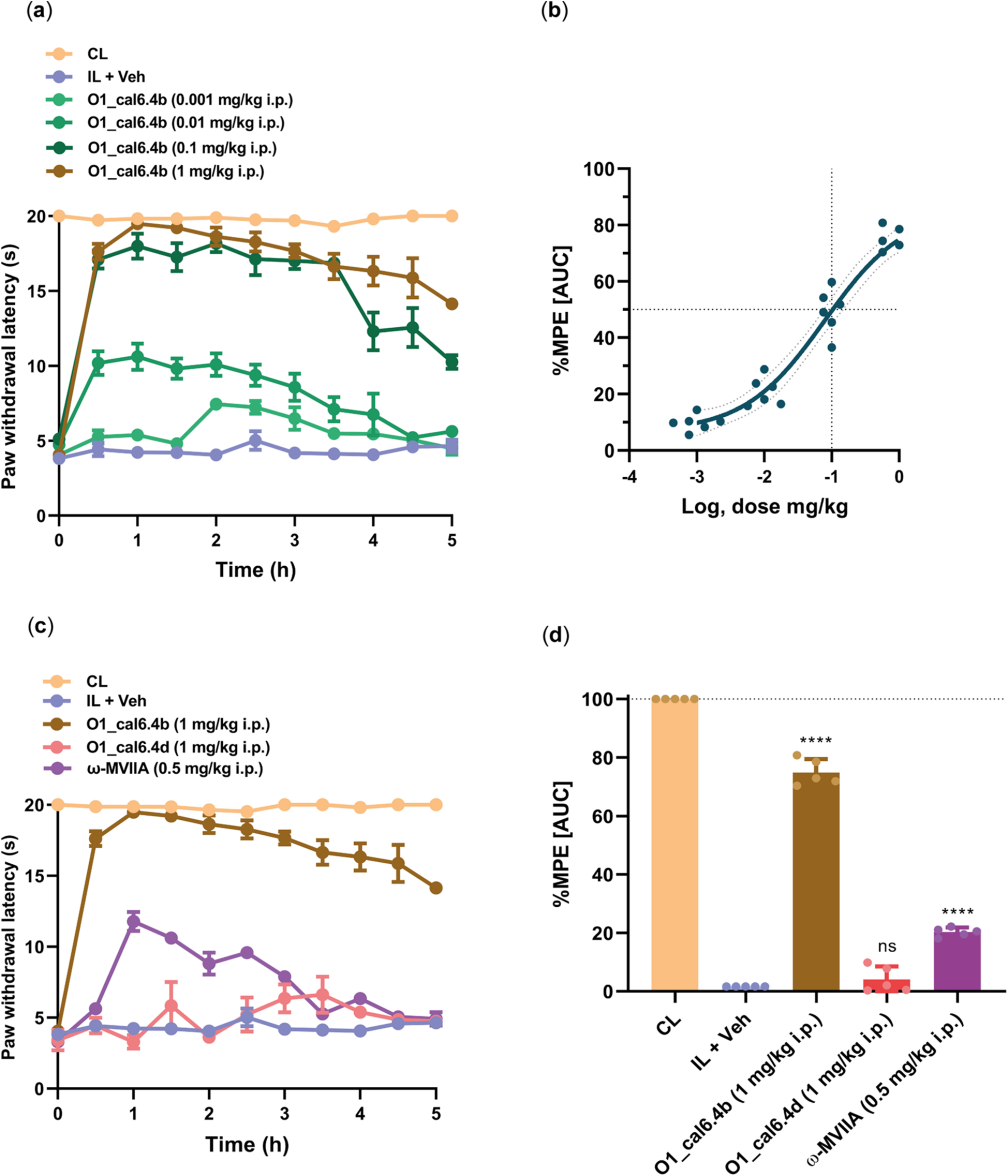
Antihyperalgesic effect of the intraperitoneal administration of O1_cal6.4b and comparison with other conotoxins in a model of inflammatory pain. (**a**) Time course of the antihyperalgesic effect of O1_cal6.4b (0.001–1 mg/kg). (**b**) Dose–response of the antihyperalgesic effect of O1_cal6.4b expressed as %MPE. An ED_50_ of 0.1 mg/kg was established with a Hill slope of 0.7303. (**c**) Comparison of the antihyperalgesic effect of the three conotoxins tested (O1_cal6.4b, O1_cal6.4d, and *ω*-MVIIA) in a model of inflammatory pain in rats. (**d**) Comparison of the antihyperalgesic effect of the three conotoxins tested (O1_cal6.4b, O1_cal6.4d, and *ω*-MVIIA) expressed as %MPE. Data are the mean ± SEM of five to six rats. ****P < 0.0001, significantly different vs. vehicle (IL + Veh) group, as determined by one-way ANOVA, followed by Dunnett's test. CL, contralateral (normal paw); IL, ipsilateral (paw with CFA); ns, non-significant; AUC, area under the curve; MPE, maximal possible effect.

To evaluate the analgesic potential of O1_cal6.4b, we employed the Hargreaves test in female rats subjected to CFA injection. Intraperitoneal administration of O1_cal6.4b ranging from 0.001 to 1 mg/kg resulted in a significant and dose-dependent reduction in CFA-induced thermal hyperalgesia (Figures 4a,b). At 1 mg/kg dose, O1_cal6.4b fully reversed thermal hyperalgesia, achieving a peak response within 1 h. This effect remained high, continuing for over 3 h (Figure 4a). In terms of %MPE, the greatest dose of O1_cal6.4b reduced thermal hyperalgesia by 74.81 ± 0.47% during the 5-h evaluation period (Figure 4b). In addition, O1_cal6.4b reduced thermal hyperalgesia in a dose-dependent manner, with an ED_50_ of 0.1 mg/kg (Hill slope: 0.7303, 95% CI: 0.31–1.41) (Figure 4b).

To compare the antihyperalgesic activity of O1_cal6.4b with other conotoxins, we administered *ω*-MVIIA (0.5 mg/kg, i.p.) and compared its effect with that of O1_cal6.4b (Figure 4c). This antihyperalgesic effect was lower than that induced by O1_cal6.4b (*P* < 0.001). Analysis of the area under the curve showed a maximal antihyperalgesic effect of O1_cal6.4b of about 75% vs. 20% for *ω*-MVIIA (*P* < 0.001) (Figure 4d). In sharp contrast, O1_cal6.4b did not induce a significant effect in the model (Figure 4d).

**Figure 5:**
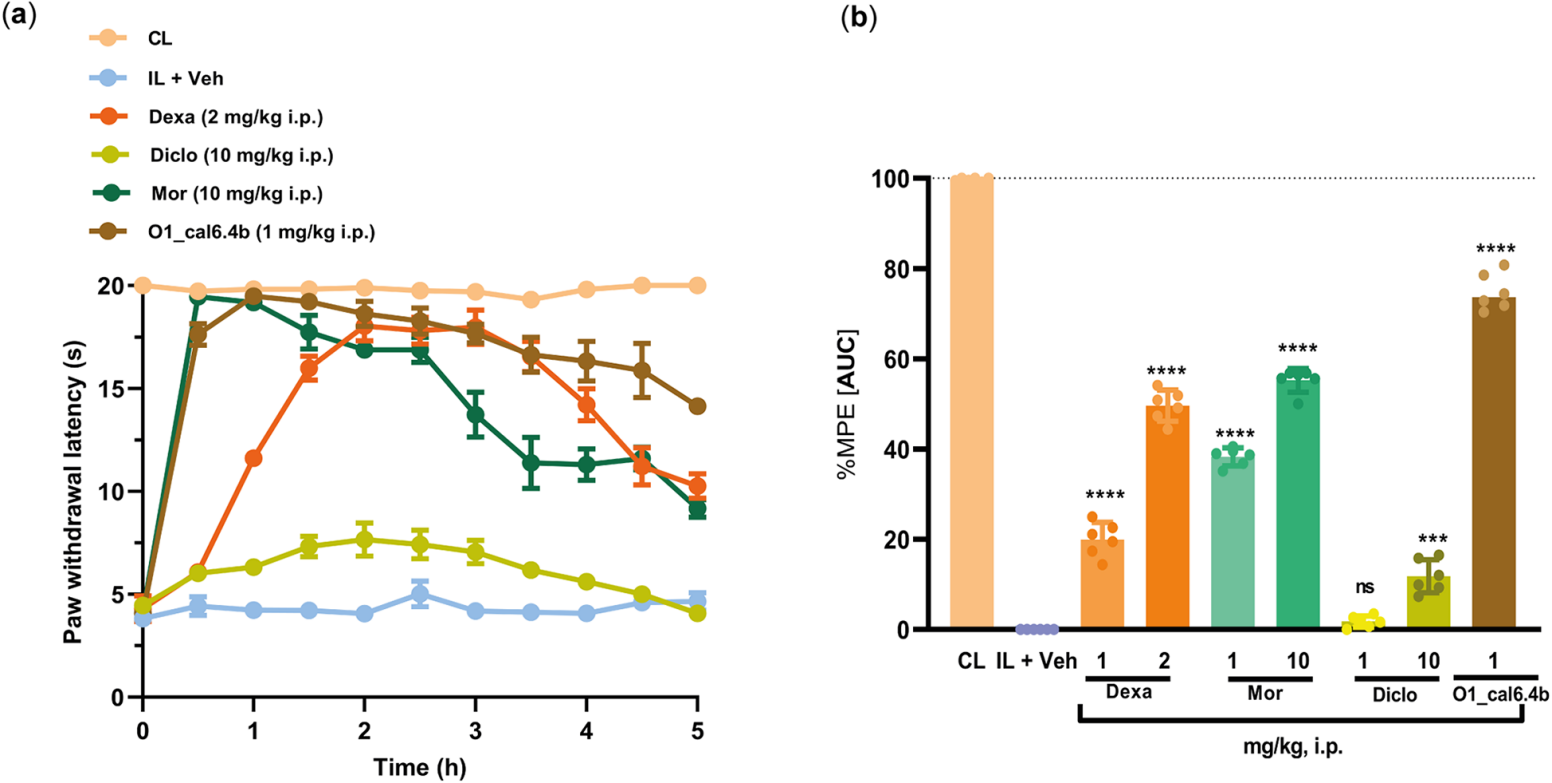
Comparison of the antihyperalgesic effect of O1_cal6.4b (1 mg/kg, i.p.) with standard analgesic drugs (dexamethasone, morphine, and diclofenac) in a model of inflammatory pain in rats. (**a**) Time course effect of the antihyperalgesic of O1_cal6.4b (1 mg/kg, i.p.) and its comparison with dexamethasone (2 mg/kg, i.p.), morphine (10 mg/kg, i.p.), and diclofenac (10 mg/kg). **(b)** Comparison of the antihyperalgesic effect of O1_cal6.4b with dexamethasone (1–2 mg/kg, i.p.), morphine (1–10 mg/kg, i.p.), and diclofenac (1–10 mg/kg, i.p.) expressed as %MPE. Data are presented as mean ± SEM of five to six animals. ****P < 0.0001 and ***P < 0.001 vs. O1_cal6.4b group, as determined by one-way ANOVA, followed by the Dunnett test. CL, contralateral (normal paw); IL, ipsilateral (paw with CFA); ns, non-significant; AUC, area under the curve; MPE, maximal possible effect; Dexa, dexamethasone; Mor, morphine; Diclo, diclofenac.

Positive controls included the steroidal anti-inflammatory drug dexamethasone (2 mg/kg, i.p.), non-steroidal anti-inflammatory drug diclofenac (10 mg/kg, i.p.), and opioid analgesic drug morphine (10 mg/kg, i.p.). O1_cal6.4b induced a greater antihyperalgesic effect than dexamethasone, morphine, and diclofenac (Figure 5a). In terms of %MPE, O1_cal6.4b (about 75%) showed a significant difference compared with dexamethasone (about 50%), morphine (about 55%), and diclofenac (about 10%) (*P* < 0.0001) (Figure 5b).

**Figure 6:**
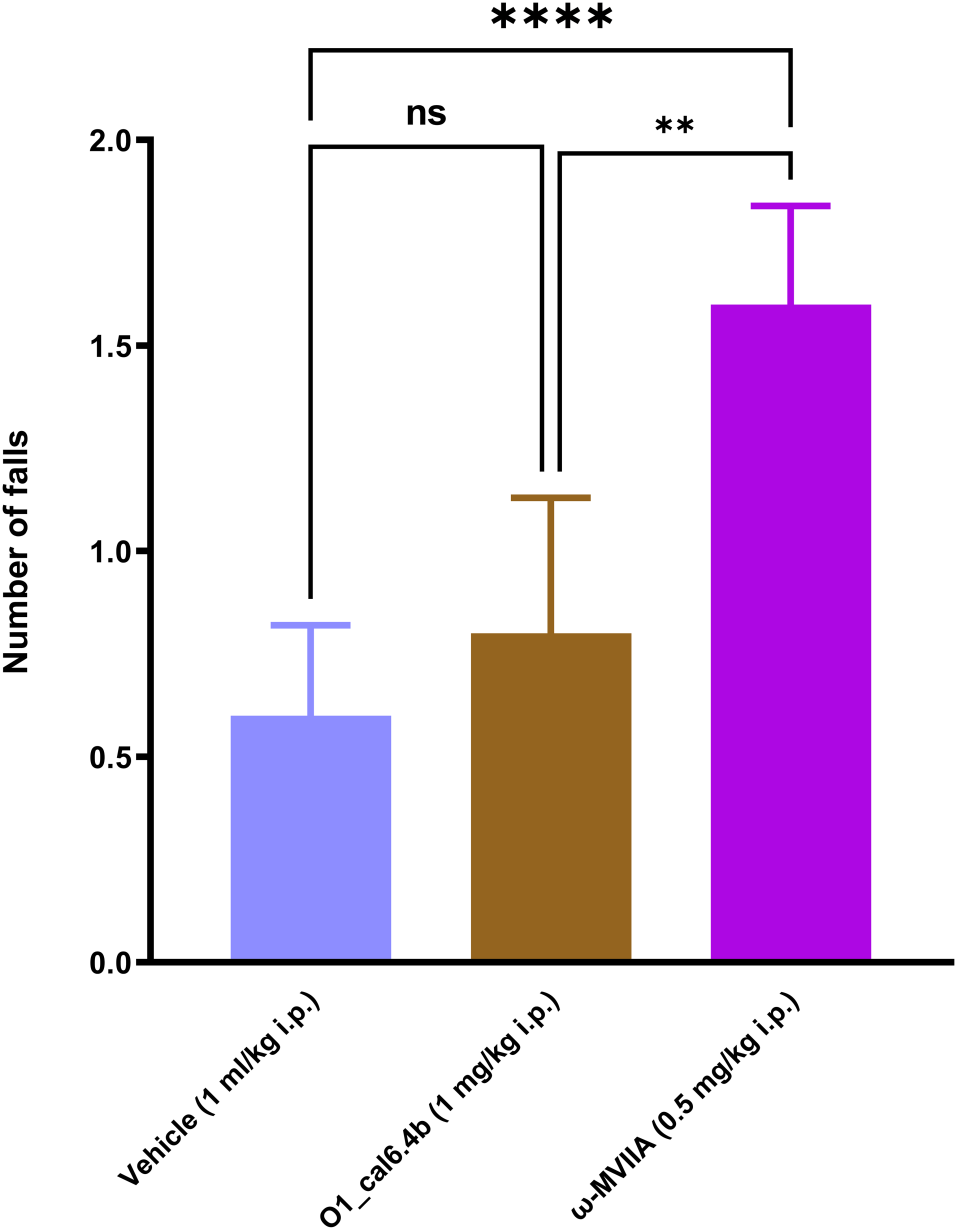
Effect of the three conotoxins tested on motor coordination in the Rota-rod test. The plot depicts the Rota-rod test results before and 1 h after O1_cal6.4b (1 mg/kg i.p.), *ω*-MVIIA (0.5 mg/kg, i.p.), or vehicle. Data are the mean ± SEM of six animals. ****P < 0.0001 and **P < 0.01 vs. Veh group, as determined by one-way ANOVA, followed by the Dunnett test. , Veh, vehicle; ns, non-significant.

According to the Rota-rod test, O1_cal6.4b (1 mg/kg i.p.) and the vehicle did not affect motor coordination. By contrast, systemic administration of *ω*-MVIIA (ziconotide) enhanced the number of falls compared with saline and O1_cal6.4b (*P* < 0.0001) (Figure 6). Unlike systemic administration of O1_cal6.4b, intraperitoneal administration of *ω*-MVIIA induced sedation, eye movements, piloerection, spontaneous tail-flick, and body tremor in some animals.

## 4 Discussion

In this study, the hydropathy analysis of conotoxins O1_cal6.4b and O1_cal6.4d provides further insights into their structural properties. Both peptides have positive average hydropathy scores, indicating a tendency toward hydrophobicity, which suggests their potential interactions with lipid membranes or hydrophobic pockets within proteins. Specifically, O1_cal6.4b has an average hydropathy score of 0.36, making it slightly more hydrophobic than O1_cal6.4d, which has a score of 0.32. This difference in hydrophobicity could influence their biological interactions and efficacy, with O1_cal6.4b potentially exhibiting enhanced membrane penetration or binding affinity to hydrophobic regions, thereby contributing to its observed antihyperalgesic effects.

The remarkable molecular diversity exhibited by conotoxins arises from a combination of factors, including the various disulfide scaffolds they employ, the extensive variability in the primary amino acid sequences located between the conserved cysteine residues, and a range of post-translational modifications (31). These adaptations have evolved in cone snails to enable conotoxins to exert their effects at both pre- and post-synaptic transmission levels, achieved through modulation of numerous ion channels and receptors; this broad range of molecular targets includes G protein-coupled receptors, neurotransmitter systems, transporters, sodium, and voltage-gated calcium channels (30, 48, 49).

Regarding the behavioral results, we observed that CFA-induced peripheral inflammation had a rapid onset of peripheral thermal hyperalgesia in both male and female rats, becoming evident approximately 4–5 h after the administration of CFA. This period corresponds to the developmental phase of persistent inflammatory pain. Furthermore, nociceptive hypersensitivity persisted for an extended duration, spanning from 24 to 72 h post-CFA injection, which corresponds to the maintenance phase of inflammatory pain. It is worth noting that thermal hyperalgesia displayed a consistent time course of %MPE between male and female rats, with no significant differences observed. These findings align with previous research conducted by other investigators, further validating the robustness and consistency of our results (50–52).

While there is a substantial body of literature on various aspects of intrathecal or intracerebroventricular administration of conotoxins, a notable gap exists in information regarding alternative routes of administration, particularly systemic injection. Previous studies have reported a lack of antinociceptive effect after intraperitoneal administration of GVIA, even at concentrations 5,000-fold higher than the ED_50_ observed with intracerebral injection (53). In addition, systemic administration of *ω*-MVIIA (ziconotide), at doses that do not induce side effects (0.02 mg/kg), is not effective in relieving mechanical allodynia in a model of diabetic neuropathy (20). These results contrast with its efficacy by the intrathecal route. Thus, the use of these drugs is limited due to factors such as intrathecal administration and a narrow therapeutic window. More recently, it has been reported that the conotoxin leconotide, at the maximum no-side-effect dose (2 mg/kg, i.v.), reaches only 52% of antinociception (20). Our data with O1_cal6.4b indicate that this conotoxin reaches about 75% of the %MPE after systemic administration without alterations in motor coordination. Although effects were obtained in different models, this information suggests the importance of the conotoxin described in the present document since it can be administered intraperitoneally, yielding a positive impact on the nociceptive response and effectively inhibiting inflammatory pain. However, the testing of O1_cal6.4b in a neuropathic pain model is necessary. A recent study evaluated the antinociceptive effect of the *ω*-conotoxin Bu8 after intracerebroventricular administration (22). Bu8 showed greater or similar antinociceptive activity than *ω*-MVIIA. However, its intramuscular administration was lethal for fishes. Data on systemic administration in rodents are lacking. Currently, there is an interest in developing small molecules derived from *ω*-conotoxins with oral bioavailability. NMED-160 advanced to phase II clinical trials. Although this drug appeared to be safe and well-tolerated in clinical trials, development was terminated without explanation (54). Another small molecule, Z160, also advanced to phase II clinical trials (30). No further information is available to date.

Interestingly, administration of O1_cal.6.4b was more effective than *ω*-MVIIA (ziconotide) as well as morphine, dexamethasone, and diclofenac. This superior effect was reached at doses that do not induce changes in motor coordination. By contrast, *ω*-MVIIA (ziconotide), at a lower dose, altered motor coordination (this study), while there is evidence that morphine (10 mg/kg) strongly affects motor coordination (55). Thus, O1_cal.6.4b has advantages over established drugs to treat inflammatory pain. Unlike O1_cal6.4b, systemic administration of O1_cal6.4d was totally ineffective in the same model. The main difference between O1_cal6.4b and O1_cal6.4d is free Arg13 in the former and bonded Arg13 in the latter. This structural feature of O1_cal.6.4b may be relevant to interact with its target leading to its antihyperalgesic effect. Another possibility to explain the difference in the antihyperalgesic effects is the differences in hydrophobicity of both conotoxins.

The mechanism by which O1_cal.6.4b induces the antihyperalgesic effect is currently unknown. There is evidence that *ω*-conotoxins are selective to block calcium voltage-gated ion channels. Most conotoxins are selective for the N-type calcium voltage-gated channel, although there are a few instances where they target the P/Q type calcium voltage-gated channel (30, 56). Since these channels are implicated in neurotransmitter release at spinal cord synapses and neurogenic inflammation in peripheral nerve endings (57), it is likely that O1_cal.6.4b may interact with these channels to induce its antihyperalgesic effect in this model. However, this will need further verification. Since *ω*-conotoxins are little permeable through the brain-blood barrier (58, 59), it is likely that the antihyperalgesic effect of O1_cal.6.4b is peripheral. Thus, this drug may act at the primary afferent neurons and at the dorsal root ganglia. This property may be relevant to avoid the adverse effects of conotoxins given by the intrathecal route.

## 5 Conclusion

In conclusion, our study reveals a potent and effective activity of O1_cal6.4b, a novel conotoxin derived from *C. californicus*, in an inflammatory pain model. Intraperitoneal administration of O1_cal6.4b induced a dose-dependent reduction of CFA-induced thermal hyperalgesia with sustained effects for about 5 h. Comparative analyses with ziconotide and other standard analgesics underscored its efficacy and unique activity profile. Interestingly, the effect of O1_cal6.4b, unlike *ω*-MVIIA, is observed at doses that do not interfere with motor coordination. The possibility of administering O1_cal6.4b intraperitoneally enhances its clinical potential, addressing gaps in alternative administration routes for conotoxins. Our findings position O1_cal6.4b as a promising candidate for innovative analgesic development in pain medicine.

## Data Availability

The datasets presented in this study can be found in online repositories. The names of the repository/repositories and accession number(s) can be found in the article/Supplementary Material.
