# Supplementary material for: Systemic antihyperalgesic effect of a novel conotoxin from Californiconus californicus in an inflammatory pain model
Source: Front Pain Res (Lausanne). 2025 Jan 24;5:1500789. doi: 10.3389/fpain.2024.1500789 (PMC11802583; doi:10.3389/fpain.2024.1500789)
Supplement: Supplementary file 1 [file Table1.docx]

**Supporting information**

**Systemic Antihyperalgesic Effect of a Novel Conotoxin from Californiconus californicus in a Chronic Inflammatory Pain Model**

Joaquín López-Carrillo^1^, Johanna Bernáldez-Sarabia^1^, Tushar Janardan Pawar^2^, Samanta Jiménez^1^, Salvador Dueñas^1^, Vinicio Granados-Soto^4^, Andrea Figueroa-Montiel^1^, José Luis Olivares-Romero^2^, Alexei F. Licea-Navarrro^1,3*^, Nadia L. Caram-Salas^1,5*^

^1^Departamento de Innovación Biomédica, CICESE, Carretera Ensenada-Tijuana No. 3918, Zona Playitas, Ensenada, C.P. 22860, Baja California, México.

^2^Red de Estudios Moleculares Avanzados, Instituto de Ecología (INECOL), Carretera antigua a Coatepec 351, Col. El Haya, C.P. 91073, Xalapa, Veracruz, Mexico.

^3^Dirección de Impulso a la Innovación y el Desarrollo (DIID), CICESE, Carretera Ensenada-Tijuana No. 3918, Zona Playitas, Ensenada, C.P. 22860, Baja California, México.

^4^Laboratorio de Neurobiología del Dolor, Departamento de Farmacobiología, CINVESTAV, Campus SUR, Calzada dde los Tenorios 235, Col. Granjas Coapa, Del Tlalpan, C.P. 14330. Ciudad de Mexico, Mexico.

^5^Investigadora por México-CONAHCYT Av. Insurgentes Sur 1582, Col. Crédito Constructor. Del. Benito Juárez, C.P. 03940, Ciudad de México, México.

*** Correspondence:**

Nadia L. Caram-Salas

[ncaram@cicese.mx](mailto:ncaram@cicese.mx)

Alexei Licea Navarro

[alicea@cicese.mx](mailto:alicea@cicese.mx)

**Affiliations**

1 *Departamento de Innovación Biomédica, CICESE, Carretera Ensenada-Tijuana No. 3918, Zona Playitas, Ensenada, C.P. 22860, Baja California, México.*

2 *Dirección de Impulso a la Innovación y el Desarrollo (DIID), CICESE, Carretera Ensenada-Tijuana No. 3918, Zona Playitas, Ensenada, C.P. 22860, Baja California, México.*

3 *Laboratorio de Neurobiología del Dolor, Departamento de Farmacobiología, CINVESTAV, Campus SUR, Calzada de los Tenorios 235, Col. Granjas Coapa, Del Tlalpan, C.P. 14330. Ciudad de México, México.*

4 *Red de Estudios Moleculares Avanzados, Instituto de Ecología (INECOL), Carretera a Coatepec 351, Col. El Haya, C.P. 91073, Xalapa, Veracruz, México.*

5 *Investigadora por México-CONAHCYT Av. Insurgentes Sur 1582, Col. Crédito Constructor. Del. Benito Juárez, C.P. 03940, Ciudad de México, México.*

**e-mail**

Dra. Nadia Caram-Salas: [ncaram@cicese.mx](mailto:ncaram@cicese.mx)

Table Index

[Table S1. Data of withdrawal latency in seconds measured in the CFA paw of female rats 4](#_Toc175131700)

[Table S2. Data of withdrawal latency in seconds measured in the normal paw of female rats 4](#_Toc175131701)

[Table S3. Data of withdrawal latency in seconds measured in the CFA paw of male rats 5](#_Toc175131702)

[Table S4. Data of withdrawal latency in seconds measured in the normal paw of female rats 5](#_Toc175131703)

[Table S5. Evaluation of Control group. Data of withdrawal latency in seconds measured in the normal paw of the animals 6](#_Toc175131704)

[Table S6. Evaluation of Saline group. Data of withdrawal latency in seconds measured in the inflamed paw of the animals 6](#_Toc175131705)

[Table S7. Evaluation of 01_cal6.4b at 0.001 mg/kg. Data of withdrawal latency in seconds measured in the inflamed paw of the animals 7](#_Toc175131706)

[Table S8. Evaluation of 01_cal6.4b at 0.01 mg/kg. Data of withdrawal latency in seconds measured in the inflamed paw of the animals 7](#_Toc175131707)

[Table S9. Evaluation of 01_cal6.4b at 0.1 mg/kg. Data of withdrawal latency in seconds measured in the inflamed paw of the animals 8](#_Toc175131708)

[Table S10. Evaluation of 01_cal6.4b at 1 mg/kg. Data of withdrawal latency in seconds measured in the inflamed paw of the animals 8](#_Toc175131709)

[Table S11. Evaluation of Dexamethasone at 2 mg/kg. Data of withdrawal latency in seconds measured in the inflamed paw of the animals 9](#_Toc175131710)

[Table S12. Evaluation of Morphine at 10 mg/kg. Data of withdrawal latency in seconds measured in the inflamed paw of the animals 9](#_Toc175131711)

[Table S13. Evaluation of w-MVIIA at 0.5 mg/kg. Data of withdrawal latency in seconds measured in the inflamed paw of the animals 10](#_Toc175131712)

[Table S14. Evaluation of 01_cal6.4d at 1 mg/kg. Data of withdrawal latency in seconds measured in the inflamed paw of the animals 10](#_Toc175131713)

[Table S15.Data of Percentage of Maximum Possible Effect (%MPE) from all the evaluated groups 11](#_Toc175131714)

[Table S16. Summary of Best-Fit values from interpolation of Standard Sigmoidal curve 11](#_Toc175131715)

[Table S17. One-way ANOVA test. Summary realized with data of %MEP 12](#_Toc175131716)

[Table S18. All data of one-way ANOVA test. Analysis realized of %MPE data 12](#_Toc175131717)

[Table S19. Dunnett’s test. Summary of data for post hoc analysis in %MPE data from all the evaluated groups 12](#_Toc175131718)

[Table S20. Tukey test. Summary of data for post hoc analysis in %MPE data from all the evaluated groups 13](#_Toc175131719)

[Table S21. One-way ANOVA test. Summary of analysis realized of %MPE data of comparison of O1_cal6.4b and O1_cal6.4d 14](#_Toc175131720)

[Table S22. One-way ANOVA parameters from comparisons of Cal6.4b and Cal6.4d 14](#_Toc175131721)

[Table S23. Dunnett´s test from comparisons of Cal6.4b and Cal6.4d 15](#_Toc175131722)

Figure Index

[Figure S1. RMSD of models a) cal6.4b and b) cal6.4d. 16](#_Toc175224882)

**1. Graphical Data: Animal Models**

*1.1. Temporal Course Data*

**Table S1.** Data of withdrawal latency in seconds measured in the CFA paw of female rats

| CFA paw | | | | | | | | | |
| --- | --- | --- | --- | --- | --- | --- | --- | --- | --- |
| Time (min) | **R1** | **R2** | **R3** | **R4** | **R5** | **R6** | **Ave** | **SEM** |  |
| 0 | 19.35 | 19.36 | 19.04 | 18.86 | 19.21 | 19.08 | 19.15 | 0.08 |  |
| 15 | 11.27 | 13.16 | 13.51 | 15.04 | 12.03 | 13.32 | 13.06 | 0.53 |  |
| 30 | 12.62 | 13.16 | 13.51 | 11.68 | 12.61 | 10.34 | 12.32 | 0.47 |  |
| 45 | 11.56 | 11.53 | 10.03 | 10.64 | 9.53 | 10.08 | 10.56 | 0.34 |  |
| 60 | 8.52 | 8.14 | 9.53 | 7.82 | 8.45 | 8.5 | 8.49 | 0.23 |  |
| 90 | 6.31 | 7.89 | 6.21 | 6.21 | 7.06 | 6.83 | 6.75 | 0.27 |  |
| 120 | 6.14 | 6.31 | 5.05 | 5.32 | 6.23 | 4.82 | 5.65 | 0.27 |  |
| 150 | 6.18 | 5.93 | 4.56 | 5.55 | 6.03 | 4.46 | 5.45 | 0.31 |  |
| 180 | 5.31 | 5.12 | 5.18 | 5.1 | 5.14 | 5.16 | 5.17 | 0.03 |  |
| 1 day | 5.1 | 5.02 | 4.21 | 4.24 | 4.44 | 4.35 | 4.56 | 0.16 |  |
| 2 days | 4.01 | 4.31 | 3.96 | 4.02 | 4.05 | 4.31 | 4.11 | 0.06 |  |
| 4 days | 4.31 | 3.92 | 3.15 | 3.19 | 3.45 | 4.35 | 4.00 | 0.22 |  |

**Table S2.** Data of withdrawal latency in seconds measured in the normal paw of female rats

| Normal Paw | | | | | | | | | |
| --- | --- | --- | --- | --- | --- | --- | --- | --- | --- |
| Time (min) | **R1** | **R2** | **R3** | **R4** | **R5** | **R6** | **Ave** | **SEM** |  |
| 0 | 19.21 | 20 | 20 | 20 | 18.11 | 20 | 19.55 | 0.32 |  |
| 15 | 20 | 20 | 20 | 20 | 20 | 20 | 20.00 | 0.00 |  |
| 30 | 20 | 20 | 20 | 20 | 20 | 19.21 | 19.87 | 0.13 |  |
| 45 | 20 | 19.36 | 19.95 | 19.04 | 19.04 | 19.5 | 19.48 | 0.17 |  |
| 60 | 20 | 20 | 18.95 | 18.32 | 18.32 | 19.04 | 19.11 | 0.31 |  |
| 90 | 19.35 | 19.52 | 20 | 19.2 | 19.2 | 19.06 | 19.39 | 0.14 |  |
| 120 | 18.82 | 18.81 | 19.38 | 18.86 | 18.32 | 18.25 | 18.74 | 0.17 |  |
| 150 | 20 | 19.52 | 20 | 18.96 | 19.23 | 18.35 | 19.34 | 0.26 |  |
| 180 | 18.86 | 20 | 18.9 | 18.08 | 18.08 | 18.24 | 18.69 | 0.30 |  |
| 1 day | 18.98 | 19.56 | 19.85 | 18.92 | 18.28 | 18.54 | 19.02 | 0.24 |  |
| 2 days | 18.56 | 19.02 | 19.52 | 19.06 | 19.35 | 18.36 | 18.98 | 0.18 |  |
| 4 days | 19.56 | 19.35 | 19.86 | 19.84 | 20 | 20 | 19.77 | 0.11 |  |

**Table S3.** Data of withdrawal latency in seconds measured in the CFA paw of male rats

| CFA paw | | | | | | | |
| --- | --- | --- | --- | --- | --- | --- | --- |
| Time (min) | **R1** | **R2** | **R3** | **R4** | **R5** | **Ave** | **SEM** |
| 0 | 19.73 | 20 | 19.56 | 18.96 | 19.4 | 19.53 | 0.17 |
| 15 | 12.36 | 13.5 | 12.82 | 13.02 | 12.02 | 12.74 | 0.26 |
| 30 | 12.08 | 13.92 | 12.95 | 12.56 | 12 | 12.70 | 0.35 |
| 45 | 11.62 | 10.44 | 10.65 | 9.96 | 9.69 | 10.47 | 0.33 |
| 60 | 8.57 | 8.55 | 8.46 | 7.32 | 7.32 | 8.04 | 0.30 |
| 90 | 7.51 | 6.1 | 6.47 | 7.31 | 6.38 | 6.75 | 0.28 |
| 120 | 6.03 | 5.96 | 6.01 | 5.44 | 5.36 | 5.76 | 0.15 |
| 150 | 5.06 | 4.58 | 5.3 | 5.15 | 5.16 | 5.05 | 0.12 |
| 180 | 4.85 | 5.07 | 5.18 | 4.36 | 5.03 | 4.90 | 0.14 |
| 1 day | 4.32 | 5.07 | 5.08 | 5.2 | 4.36 | 4.81 | 0.19 |
| 2 days | 4.06 | 4.58 | 4.3 | 5.05 | 5.16 | 4.63 | 0.21 |
| 4 days | 4.31 | 4.21 | 5.03 | 4.33 | 3.86 | 4.00 | 0.19 |

**Table S4.** Data of withdrawal latency in seconds measured in the normal paw of female rats

| CFA paw | | | | | | | |
| --- | --- | --- | --- | --- | --- | --- | --- |
| Time | **R1** | **R2** | **R3** | **R4** | **R5** | Ave | SEM |
| 0 | 19.73 | 20 | 19.59 | 20 | 18.11 | 19.49 | 0.35 |
| 15 | 20 | 20 | 20 | 20 | 20 | 20.00 | 0.00 |
| 30 | 18.56 | 18.91 | 20 | 20 | 20 | 19.49 | 0.31 |
| 45 | 18.94 | 19.69 | 18.26 | 19.95 | 18.96 | 19.16 | 0.30 |
| 60 | 20 | 18.37 | 18.35 | 20 | 20 | 19.34 | 0.40 |
| 90 | 20 | 18.51 | 19.32 | 20 | 20 | 19.57 | 0.30 |
| 120 | 18.65 | 19.23 | 20 | 19.82 | 17.99 | 19.14 | 0.37 |
| 150 | 20 | 18.96 | 18.57 | 19.32 | 19.51 | 19.27 | 0.24 |
| 180 | 20 | 18.96 | 18.57 | 19.32 | 19.51 | 19.27 | 0.24 |
| 1 day | 18.98 | 20 | 20 | 18.92 | 19.56 | 19.49 | 0.24 |
| 2 days | 20 | 19.02 | 19.52 | 20 | 19.35 | 19.58 | 0.19 |
| 4 days | 19.56 | 20 | 18.97 | 18.96 | 20 | 19.50 | 0.23 |

**Table S5.** Evaluation of Control group. Data of withdrawal latency in seconds measured in the normal paw of the animals

| **Normal paw** | | | |  | | | | | | |
| --- | --- | --- | --- | --- | --- | --- | --- | --- | --- | --- |
| **Time (min)** | **R1** | **R2** | **R3** | | **R4** | **R5** | **R6** | **Ave** | **SEM** |  |
| **0** | 20 | 20 | 20 | | 19.87 | 19.56 | 20 | 19.91 | 0.07 |  |
| **30** | 20 | 20 | 20 | | 20 | 20 | 20 | 20.00 | 0.00 |  |
| **60** | 20 | 20 | 19.85 | | 20 | 20 | 19.95 | 19.97 | 0.02 |  |
| **90** | 20 | 20 | 19.36 | | 19.43 | 19.87 | 19.99 | 19.78 | 0.12 |  |
| **120** | 20 | 20 | 20 | | 20 | 18.56 | 20 | 19.76 | 0.24 |  |
| **150** | 18.52 | 19.35 | 19.96 | | 20 | 20 | 19.02 | 19.48 | 0.25 |  |
| **180** | 19.52 | 19.11 | 19.01 | | 20 | 20 | 19.7 | 19.56 | 0.17 |  |
| **210** | 19.69 | 19.31 | 19.56 | | 20 | 20 | 19.99 | 19.76 | 0.12 |  |
| **240** | 19.32 | 19.43 | 19.44 | | 19.56 | 20 | 20 | 19.63 | 0.12 |  |
| **270** | 20 | 20 | 20 | | 20 | 20 | 20 | 20.00 | 0.00 |  |
| **300** | 20 | 20 | 20 | | 20 | 20 | 20 | 20.00 | 0.00 |  |
|  |  |  |  | |  |  |  |  |  |  |
| **AUC** | 5301.3 | 5307.45 | 5308.13 | | 5362.13 | 5349.6 | 5359.88 | 5331.41 | 11.70 |  |

**Table S6.** Evaluation of Saline group. Data of withdrawal latency in seconds measured in the inflamed paw of the animals

| **CFA Paw** | | | | |  | | | | |
| --- | --- | --- | --- | --- | --- | --- | --- | --- | --- |
| **Time (min)** | **R1** | **R2** | **R3** | **R4** | | **R5** | **R6** | **Ave** | **SEM** |
| **0** | 19.22 | 18.99 | 19.72 | 19.21 | | 20 | 20 | 19.52 | 0.18 |
| **30** | 10.99 | 17.41 | 17.6 | 17.89 | | 16.98 | 18.45 | 16.55 | 1.13 |
| **60** | 5.69 | 7.95 | 12.22 | 9.18 | | 9.31 | 10.67 | 9.17 | 0.92 |
| **90** | 5.69 | 9.2 | 10.19 | 7.63 | | 8.22 | 8.41 | 8.22 | 0.62 |
| **120** | 4.28 | 3.91 | 3.87 | 3.53 | | 3.7 | 3.99 | 3.88 | 0.10 |
| **150** | 4.66 | 3.98 | 3.65 | 3.87 | | 3.27 | 4.01 | 3.91 | 0.19 |
| **180** | 5.36 | 4.31 | 3.91 | 3.52 | | 4.01 | 4.03 | 4.19 | 0.26 |
| **210** | 4.89 | 4.33 | 4.01 | 4.05 | | 3.87 | 3.65 | 4.13 | 0.18 |
| **240** | 3.89 | 4.99 | 3.65 | 3.87 | | 3.99 | 4.03 | 4.07 | 0.19 |
| **270** | 3.87 | 3.89 | 4.78 | 4.95 | | 5.01 | 5.03 | 4.59 | 0.23 |
| **300** | 3.55 | 5.91 | 3.76 | 4.67 | | 3.76 | 3.9 | 4.26 | 0.37 |
|  |  |  |  |  | |  |  |  |  |
| **AUC** | 1474.575 | 1695.6 | 1763.4 | 1619.63 | | 1636.13 | 1721.78 | 1638.30 | 61.97 |

**Table S7.** Evaluation of 01_cal6.4b at 0.001 mg/kg. Data of withdrawal latency in seconds measured in the inflamed paw of the animals

| **CFA Paw** | | | | |  | | | | | |
| --- | --- | --- | --- | --- | --- | --- | --- | --- | --- | --- |
| **Time (min)** | **R1** | **R2** | **R3** | **R4** | | **R5** | **R6** | **Ave** | **SEM** |  |
| **0** | 3.38 | 3.77 | 4.9 | 3.89 | | 3.45 | 5.44 | 4.14 | 0.34 |  |
| **30** | 6.78 | 8.98 | 7.22 | 8.33 | | 6.98 | 6.43 | 5.01 | 0.40 |  |
| **60** | 5.67 | 7.34 | 6.89 | 7.65 | | 8.9 | 6.98 | 6.90 | 0.43 |  |
| **90** | 3.29 | 6.09 | 6.98 | 6.43 | | 8.9 | 7.21 | 5.98 | 0.75 |  |
| **120** | 4.87 | 6.33 | 5.77 | 3.87 | | 7.89 | 6.43 | 5.42 | 0.57 |  |
| **150** | 5.88 | 5.9 | 5.71 | 5.98 | | 6.87 | 5.91 | 5.33 | 0.17 |  |
| **180** | 7.98 | 4.76 | 5.9 | 6.92 | | 5.87 | 6.32 | 5.04 | 0.44 |  |
| **210** | 4.67 | 5.87 | 6.02 | 6.19 | | 5.1 | 5.01 | 5.48 | 0.26 |  |
| **240** | 5.78 | 4.78 | 4.87 | 5.98 | | 6.02 | 5.33 | 5.46 | 0.22 |  |
| **270** | 4.71 | 3.87 | 4.95 | 4.91 | | 5.87 | 5.98 | 3.89 | 0.32 |  |
| **300** | 3.65 | 3.09 | 5.89 | 4.77 | | 5.32 | 4.22 | 4.49 | 0.43 |  |
|  |  |  |  |  | |  |  |  |  |  |
| **AUC** | 1594.35 | 1720.5 | 1791.15 | 1817.7 | | 2003.55 | 1812.9 | 1790.03 | 54.77 |  |

**Table S8.** Evaluation of 01_cal6.4b at 0.01 mg/kg. Data of withdrawal latency in seconds measured in the inflamed paw of the animals

| **CFA Paw** | | | | |  | | | | |
| --- | --- | --- | --- | --- | --- | --- | --- | --- | --- |
| **Time (min)** | **R1** | **R2** | **R3** | **R4** | | **R5** | **R6** | **Ave** | **SEM** |
| **0** | 3.38 | 5.29 | 4.9 | 3.78 | | 4.31 | 5.44 | 4.52 | 0.34 |
| **30** | 12.43 | 9.71 | 9.02 | 8.95 | | 10.52 | 10.23 | 10.14 | 0.52 |
| **60** | 13.32 | 9.31 | 8.76 | 9.43 | | 10.93 | 9.8 | 10.26 | 0.68 |
| **90** | 11.91 | 9.42 | 9.45 | 7.77 | | 11.99 | 11.32 | 10.31 | 0.69 |
| **120** | 11.32 | 6.88 | 7.44 | 6.49 | | 9.43 | 10.31 | 8.65 | 0.81 |
| **150** | 10.01 | 7.91 | 6.51 | 8.76 | | 8.41 | 9.31 | 8.49 | 0.49 |
| **180** | 8.88 | 6.28 | 7.29 | 6.32 | | 7.42 | 7.43 | 7.27 | 0.39 |
| **210** | 7.43 | 6.31 | 6.81 | 6.98 | | 6.29 | 6.37 | 6.70 | 0.19 |
| **240** | 5.41 | 3.43 | 5.17 | 7.02 | | 5.33 | 6.03 | 5.40 | 0.48 |
| **270** | 4.72 | 4.98 | 4.87 | 6.78 | | 4.28 | 5.21 | 5.14 | 0.35 |
| **300** | 3.43 | 4.21 | 4.33 | 4.31 | | 5.33 | 5.02 | 4.44 | 0.27 |
|  |  |  |  |  | |  |  |  |  |
| **AUC** | 2665.05 | 2069.4 | 2098.05 | 2176.35 | | 2382.6 | 2437.2 | 2304.78 | 94.52 |

**Table S9.** Evaluation of 01_cal6.4b at 0.1 mg/kg. Data of withdrawal latency in seconds measured in the inflamed paw of the animals

| **CFA Paw** | | | | |  | | | | |
| --- | --- | --- | --- | --- | --- | --- | --- | --- | --- |
| **Time (min)** | **R1** | **R2** | **R3** | **R4** | | **R5** | **R6** | **Ave** | **SEM** |
| **0** | 3.77 | 4.66 | 5.31 | 4.23 | | 3.33 | 4.32 | 4.27 | 0.28 |
| **30** | 12.32 | 19.32 | 19.2 | 18.78 | | 17.89 | 19.32 | 17.81 | 1.12 |
| **60** | 15.91 | 20 | 18.2 | 19.21 | | 15.21 | 17.33 | 17.64 | 0.76 |
| **90** | 11.74 | 18.97 | 17.21 | 16.56 | | 13.77 | 18.51 | 16.13 | 1.15 |
| **120** | 11.66 | 15.46 | 16.52 | 10.98 | | 14.35 | 17.16 | 14.36 | 1.04 |
| **150** | 10.8 | 10.21 | 17.6 | 13.28 | | 12.43 | 12.44 | 12.79 | 1.07 |
| **180** | 9.31 | 9.33 | 13.21 | 10.21 | | 9.38 | 13.7 | 10.86 | 0.84 |
| **210** | 9.47 | 10.21 | 11.42 | 10.57 | | 10.02 | 12.65 | 10.72 | 0.47 |
| **240** | 7.36 | 8.32 | 9.23 | 8.76 | | 9.52 | 6.43 | 8.27 | 0.48 |
| **270** | 6.87 | 6.92 | 8.76 | 7.32 | | 7.26 | 5.76 | 7.15 | 0.40 |
| **300** | 6.88 | 6.49 | 4.88 | 4.59 | | 6.01 | 5.03 | 5.65 | 0.39 |
|  |  |  |  |  | |  |  |  |  |
| **AUC** | 3022.95 | 3729.45 | 4093.35 | 3602.4 | | 3435 | 3839.25 | 3620.40 | 150.01 |

**Table S10.** Evaluation of 01_cal6.4b at 1 mg/kg. Data of withdrawal latency in seconds measured in the inflamed paw of the animals

| **CFA Paw** | | | | |  | | | | |
| --- | --- | --- | --- | --- | --- | --- | --- | --- | --- |
| **Time (min)** | **R1** | **R2** | **R3** | **R4** | | **R5** | **R6** | **Ave** | **SEM** |
| **0** | 4.51 | 3.82 | 3.51 | 4.37 | | 2.99 | 3.03 | 3.71 | 0.27 |
| **30** | 18.31 | 17.44 | 17.32 | 16.32 | | 20 | 20 | 18.23 | 0.62 |
| **60** | 20 | 20 | 20 | 20 | | 20 | 19.82 | 19.97 | 0.03 |
| **90** | 20 | 20 | 19.33 | 17.42 | | 18.43 | 18.36 | 18.92 | 0.42 |
| **120** | 20 | 17.03 | 16.32 | 17.51 | | 17.51 | 20 | 18.06 | 0.64 |
| **150** | 19.76 | 16.82 | 16.21 | 16.57 | | 13.37 | 20 | 17.12 | 1.01 |
| **180** | 18.98 | 20 | 20 | 20 | | 20 | 20 | 19.83 | 0.17 |
| **210** | 17.67 | 20 | 16.81 | 13.66 | | 20 | 13.7 | 16.97 | 1.16 |
| **240** | 15.35 | 15.87 | 13.22 | 13.87 | | 11.24 | 12.65 | 13.70 | 0.70 |
| **270** | 12.88 | 13.89 | 11.65 | 10.8 | | 10.99 | 9.76 | 11.66 | 0.61 |
| **300** | 7.32 | 4.81 | 5.03 | 8.89 | | 7.32 | 6.43 | 6.63 | 0.63 |
|  |  |  |  |  | |  |  |  |  |
| **AUC** | 5065.95 | 4960.95 | 4653.9 | 4583.4 | | 4700.85 | 4770.6 | 4789.28 | 76.35 |

**Table S11.** Evaluation of Dexamethasone at 2 mg/kg. Data of withdrawal latency in seconds measured in the inflamed paw of the animals

| **CFA Paw** | | | | |  | | | | |
| --- | --- | --- | --- | --- | --- | --- | --- | --- | --- |
| **Time (min)** | **R1** | **R2** | **R3** | **R4** | | **R5** | **R6** | **Ave** | **SEM** |
| **0** | 5.17 | 6.56 | 4.3 | 2.31 | | 4.54 | 2.93 | 4.30 | 0.63 |
| **30** | 7.17 | 6.91 | 5.36 | 5.32 | | 5.41 | 6.32 | 6.08 | 0.34 |
| **60** | 12.8 | 11.86 | 10.51 | 11.32 | | 10.97 | 12.18 | 11.61 | 0.34 |
| **90** | 16.31 | 14.77 | 13.23 | 14.31 | | 13.44 | 14.34 | 14.40 | 0.45 |
| **120** | 18.21 | 15.73 | 16.73 | 17.31 | | 20 | 20 | 18.00 | 0.71 |
| **150** | 19.21 | 16.33 | 19.24 | 16.43 | | 18.31 | 20 | 18.25 | 0.63 |
| **180** | 15.34 | 15.87 | 17.41 | 19.21 | | 20 | 20 | 17.97 | 0.84 |
| **210** | 15.94 | 14.31 | 14.98 | 8.99 | | 13.21 | 12.98 | 13.40 | 0.99 |
| **240** | 10.23 | 9.45 | 7.64 | 9.36 | | 8.72 | 7.98 | 8.90 | 0.40 |
| **270** | 6.9 | 9.07 | 7.88 | 7.78 | | 7.92 | 6.91 | 7.74 | 0.33 |
| **300** | 6.22 | 5.21 | 4.43 | 3.52 | | 5.21 | 4.37 | 4.83 | 0.38 |
|  |  |  |  |  | |  |  |  |  |
| **AUC** | 3834.15 | 3605.55 | 3520.35 | 3388.35 | | 3685.65 | 3730.80 | 3627.48 | 64.78 |

**Table S12**. Evaluation of Morphine at 10 mg/kg. Data of withdrawal latency in seconds measured in the inflamed paw of the animals

| **CFA Paw** | | | | |  | | | | |
| --- | --- | --- | --- | --- | --- | --- | --- | --- | --- |
| **Time (min)** | **R1** | **R2** | **R3** | **R4** | | **R5** | **R6** | **Ave** | **SEM** |
| **0** | 3.45 | 4.35 | 4.75 | 3.45 | | 4.35 | 4.15 | 4.08 | 0.22 |
| **30** | 20 | 20 | 18 | 20 | | 20 | 18.69 | 19.45 | 0.36 |
| **60** | 19 | 19.25 | 19.95 | 19 | | 19.25 | 18.6 | 19.18 | 0.18 |
| **90** | 16.01 | 15.94 | 20 | 17.99 | | 15.94 | 18.56 | 17.41 | 0.70 |
| **120** | 20 | 16.89 | 16.81 | 16.89 | | 16.89 | 17.31 | 17.47 | 0.51 |
| **150** | 18.87 | 18.57 | 17.67 | 17.87 | | 15.94 | 19.02 | 17.99 | 0.47 |
| **180** | 16.18 | 13.56 | 12.66 | 17.56 | | 10.56 | 11.85 | 13.73 | 1.09 |
| **210** | 9.42 | 12.07 | 10.99 | 6.07 | | 10.07 | 12.3 | 10.15 | 0.93 |
| **240** | 4.23 | 4.31 | 5.98 | 5.98 | | 4.31 | 5.75 | 5.09 | 0.36 |
| **270** | 4.76 | 4.89 | 4.9 | 5.01 | | 4.02 | 5.33 | 4.82 | 0.18 |
| **300** | 3.56 | 4.81 | 4.24 | 4.24 | | 4.81 | 4.88 | 4.42 | 0.21 |
|  |  |  |  |  | |  |  |  |  |
| **AUC** | 3959.25 | 3901.8 | 3943.65 | 3906.45 | | 3646.8 | 3957.75 | 3885.95 | 48.89 |

**Table S13.** Evaluation of w-MVIIA at 0.5 mg/kg. Data of withdrawal latency in seconds measured in the inflamed paw of the animals

| **CFA Paw** | | | | |  | | | | |
| --- | --- | --- | --- | --- | --- | --- | --- | --- | --- |
| **Time (min)** | **R1** | **R2** | **R3** | **R4** | | **R5** | **R6** | **Ave** | **SEM** |
| **0** | 3.52 | 3.22 | 4.23 | 3.89 | | 2.01 | 2.97 | 3.31 | 0.32 |
| **30** | 5.44 | 5.23 | 5.98 | 6.21 | | 5.6 | 5.33 | 5.63 | 0.16 |
| **60** | 12.95 | 9.21 | 10.32 | 12.89 | | 12.69 | 12.63 | 11.78 | 0.66 |
| **90** | 10.98 | 10.65 | 11.56 | 10.98 | | 9.85 | 9.66 | 10.61 | 0.30 |
| **120** | 10.98 | 9.56 | 10.56 | 8.33 | | 6.98 | 6.43 | 8.81 | 0.77 |
| **150** | 9.32 | 8.98 | 9.84 | 9.56 | | 9.82 | 9.99 | 9.59 | 0.16 |
| **180** | 7.98 | 7.26 | 7.98 | 8 | | 8.9 | 7.21 | 7.89 | 0.25 |
| **210** | 5.87 | 5.21 | 5.33 | 5.01 | | 5.1 | 5.01 | 5.26 | 0.13 |
| **240** | 6.32 | 6.98 | 7.21 | 5.98 | | 6.2 | 5.33 | 6.34 | 0.28 |
| **270** | 3.87 | 4.71 | 4.95 | 4.91 | | 5.87 | 5.98 | 5.05 | 0.32 |
| **300** | 3.09 | 6.21 | 5.89 | 4.77 | | 5.32 | 4.22 | 4.92 | 0.47 |
|  |  |  |  |  | |  |  |  |  |
| **AUC** | 2310.45 | 2175.15 | 2363.7 | 2286 | | 2240.25 | 2134.95 | 2251.75 | 35.02 |

**Table S14.** Evaluation of 01_cal6.4d at 1 mg/kg. Data of withdrawal latency in seconds measured in the inflamed paw of the animals

| **CFA Paw** | | | |  | | | |
| --- | --- | --- | --- | --- | --- | --- | --- |
| **Time (min)** | **R1** | **R2** | **R3** | **R4** | **R5** | **Ave** | **SEM** |
| **0** | 1.44 | 2.11 | 5.41 | 4.28 | 3.80 | 3.41 | 0.72 |
| **30** | 4.08 | 2.84 | 3.94 | 5.36 | 5.96 | 4.44 | 0.55 |
| **60** | 2.15 | 2.96 | 4.98 | 2.83 | 3.51 | 3.29 | 0.48 |
| **90** | 5.54 | 12.03 | 3.06 | 2.84 | 5.74 | 5.84 | 1.66 |
| **120** | 2.90 | 4.95 | 3.65 | 2.83 | 3.81 | 3.63 | 0.38 |
| **150** | 3.59 | 2.20 | 9.11 | 4.68 | 6.54 | 5.22 | 1.20 |
| **180** | 6.28 | 3.74 | 8.82 | 4.66 | 8.31 | 6.36 | 0.99 |
| **210** | 7.38 | 3.56 | 9.90 | 3.76 | 8.44 | 6.61 | 1.27 |
| **240** | 5.29 | 4.67 | 6.69 | 5.11 | 5.13 | 5.38 | 0.34 |
| **270** | 5.24 | 4.92 | 4.69 | 4.79 | 4.68 | 4.86 | 0.10 |
| **300** | 4.53 | 4.46 | 4.46 | 5.02 | 5.29 | 4.75 | 0.17 |
|  |  |  |  |  |  |  |  |
| AUC | **1362.9** | **1354.6** | **1792.9** | **1245.7** | **1699.4** | **1491.1** | 107.18 |

- 1. Maximum Possible Effect Percentage Data

**Table S15.** Data of Percentage of Maximum Possible Effect (%MPE) from all the evaluated groups

|  | **% MPE** | | | | | |  |  |
| --- | --- | --- | --- | --- | --- | --- | --- | --- |
| **Group** | **R1** | **R2** | **R3** | **R4** | **R5** | **R6** | **Ave** | **SEM** |
| **Normal Paw** | 99.49 | 100 | 99.16 | 101.6 | 99.59 | 100.48 | **100** | 0.61 |
| **Saline** | 0.00 | 0.00 | 0.00 | 0.00 | 0.00 | 0.00 | **0.00** | 0.58 |
| **O1_cal6.4b** (0.001 mg/kg) | 5.53 | 8.27 | 9.80 | 10.38 | 14.41 | 10.27 | **5.32** | 1.29 |
| **O1_cal6.4b**  (0.01 mg/kg) | 28.75 | 15.83 | 16.45 | 18.15 | 22.62 | 23.81 | **20.94** | 0.91 |
| **O1_cal6.4b**  (0.1 mg/kg) | 36.51 | 51.83 | 59.72 | 49.07 | 45.44 | 54.21 | **49.46** | 0.63 |
| **O1_cal6.4b** (1 mg/kg) | 80.81 | 78.53 | 71.87 | 70.35 | 72.89 | 74.41 | **74.81** | 0.91 |
| **O1_cal6.4d**  (1 mg/kg) | 0.51 | 0.33 | 9.84 | 2.03 | 7.81 | - | **3.29** | 0.47 |
| **Dexamethasone**  (2 mg/kg) | 54.10 | 49.14 | 47.30 | 44.43 | 50.88 | 51.86 | **49.62** | 0.63 |
| **Morphine**  (10 mg/kg) | 56.81 | 55.57 | 56.47 | 55.67 | 50.04 | 56.78 | **55.22** | 1.95 |
| **ω-MVIIA**  (0.5 mg/kg) | 18.13 | 21.06 | 22.21 | 20.53 | 19.54 | 17.25 | **19.66** | 0.00 |

**Table S16.** Summary of Best-Fit values from interpolation of Standard Sigmoidal curve

| **Best-fit values** |  |
| --- | --- |
| Top | 85.48 |
| Bottom | 6.661 |
| LogIC50 | -1.103 |
| HillSlope | 0.7303 |
| IC50 | 0.07896 |

1. **Statistical test**

*2.1. ANOVA test*

**Table S17.** One-way ANOVA test. Summary realized with data of %MEP

| F | 419.2 |
| --- | --- |
| P value | <0.0001 |
| P value summary | **** |
| Significant diff. among means (P < 0.05)? | Yes |
| R squared | 0.9868 |

**Table S18.** All data of one-way ANOVA test. Analysis realized of %MPE data

| **ANOVA table** | **SS** | **DF** | **MS** | **F (DFn, DFd)** | **P value** |
| --- | --- | --- | --- | --- | --- |
| Treatment (between groups) | 50607 | 8 | 6326 | F (8, 45) = 419.2 | P<0.0001 |
| Residual (within groups) | 679.0 | 45 | 15.09 |  |  |
| Total | 51286 | 53 |  |  |  |

- 1. *Multiple comparisons test*

**Table S19**. Dunnett’s test. Summary of data for post hoc analysis in %MPE data from all the evaluated groups

| **Dunnett’s multiple comparisons test** | **Below threshold?** | **Adjusted P Value** |
| --- | --- | --- |
| Saline vs. Normal Paw | Yes | <0.0001 |
| Saline vs. Dexamethasone (2 mg/kg i.p.) | Yes | <0.0001 |
| Saline vs. Morphine (10 mg/kg i.p.) | Yes | <0.0001 |
| Saline vs. O1_cal6.4b (1 mg/kg i.p.) | Yes | <0.0001 |
| Saline vs. O1_cal6.4b (0.1 mg/kg i.p.) | Yes | <0.0001 |
| Saline vs. O1_cal6.4b (0.01 mg/kg i.p.) | Yes | <0.0001 |
| Saline vs. O1_cal6.4b (0.001 mg/kg i.p.) | Yes | 0.0006 |
| Saline vs. ω-MVIIA (0.5 mg/kg i.p.) | Yes | <0.0001 |

**Table S20.** Tukey test. Summary of data for post hoc analysis in %MPE data from all the evaluated groups

| **Tukey's multiple comparisons test** | **Below threshold?** | **Adjusted P Value** |
| --- | --- | --- |
| Saline vs. Normal Paw | Yes | <0.0001 |
| Saline vs. Dexamethasone (2 mg/kg i.p.) | Yes | <0.0001 |
| Saline vs. Morphine (10 mg/kg i.p.) | Yes | <0.0001 |
| Saline vs. O1_cal6.4b (1 mg/kg i.p.) | Yes | <0.0001 |
| Saline vs. O1_cal6.4b (0.1 mg/kg i.p.) | Yes | <0.0001 |
| Saline vs. O1_cal6.4b (0.01 mg/kg i.p.) | Yes | <0.0001 |
| Saline vs. O1_cal6.4b (0.001 mg/kg i.p.) | Yes | 0.0022 |
| Saline vs. ω-MVIIA (0.5 mg/kg i.p.) | Yes | <0.0001 |
| Normal Paw vs. Dexamethasone (2 mg/kg i.p.) | Yes | <0.0001 |
| Normal Paw vs. Morphine (10 mg/kg i.p.) | Yes | <0.0001 |
| Normal Paw vs. O1_cal6.4b (1 mg/kg i.p.) | Yes | <0.0001 |
| Normal Paw vs. O1_cal6.4b (0.1 mg/kg i.p.) | Yes | <0.0001 |
| Normal Paw vs. O1_cal6.4b (0.01 mg/kg i.p.) | Yes | <0.0001 |
| Normal Paw vs. O1_cal6.4b (0.001 mg/kg i.p.) | Yes | <0.0001 |
| Normal Paw vs. ω-MVIIA (0.5 mg/kg i.p.) | Yes | <0.0001 |
| Dexamethasone (2 mg/kg i.p.) vs. Morphine (10 mg/kg i.p.) | No | 0.2597 |
| Dexamethasone (2 mg/kg i.p.) vs. O1_cal6.4b (1 mg/kg i.p.) | Yes | <0.0001 |
| Dexamethasone (2 mg/kg i.p.) vs. O1_cal6.4b (0.1 mg/kg i.p.) | No | >0.9999 |
| Dexamethasone (2 mg/kg i.p.) vs. O1_cal6.4b (0.01 mg/kg i.p.) | Yes | <0.0001 |
| Dexamethasone (2 mg/kg i.p.) vs. O1_cal6.4b (0.001 mg/kg i.p.) | Yes | <0.0001 |
| Dexamethasone (2 mg/kg i.p.) vs. ω-MVIIA (0.5 mg/kg i.p.) | Yes | <0.0001 |
| Morphine (10 mg/kg i.p.) vs. O1_cal6.4b (1 mg/kg i.p.) | Yes | <0.0001 |
| Morphine (10 mg/kg i.p.) vs. O1_cal6.4b (0.1 mg/kg i.p.) | No | 0.2286 |
| Morphine (10 mg/kg i.p.) vs. O1_cal6.4b (0.01 mg/kg i.p.) | Yes | <0.0001 |
| Morphine (10 mg/kg i.p.) vs. O1_cal6.4b (0.001 mg/kg i.p.) | Yes | <0.0001 |
| Morphine (10 mg/kg i.p.) vs. ω-MVIIA (0.5 mg/kg i.p.) | Yes | <0.0001 |
| O1_cal6.4b (1 mg/kg i.p.) vs. O1_cal6.4b (0.1 mg/kg i.p.) | Yes | <0.0001 |
| O1_cal6.4b (1 mg/kg i.p.) vs. O1_cal6.4b (0.01 mg/kg i.p.) | Yes | <0.0001 |
| O1_cal6.4b (1 mg/kg i.p.) vs. O1_cal6.4b (0.001 mg/kg i.p.) | Yes | <0.0001 |
| O1_cal6.4b (1 mg/kg i.p.) vs. ω-MVIIA (0.5 mg/kg i.p.) | Yes | <0.0001 |
| O1_cal6.4b (0.1 mg/kg i.p.) vs. O1_cal6.4b (0.01 mg/kg i.p.) | Yes | <0.0001 |
| O1_cal6.4b (0.1 mg/kg i.p.) vs. O1_cal6.4b (0.001 mg/kg i.p.) | Yes | <0.0001 |
| O1_cal6.4b (0.1 mg/kg i.p.) vs. ω-MVIIA (0.5 mg/kg i.p.) | Yes | <0.0001 |
| O1_cal6.4b (0.01 mg/kg i.p.) vs. O1_cal6.4b (0.001 mg/kg i.p.) | Yes | 0.0003 |
| O1_cal6.4b (0.01 mg/kg i.p.) vs. ω-MVIIA (0.5 mg/kg i.p.) | No | 0.9999 |
| O1_cal6.4b (0.001 mg/kg i.p.) vs. ω-MVIIA (0.5 mg/kg i.p.) | Yes | 0.0016 |

**Table S21.** One-way ANOVA test. Summary of analysis realized of %MPE data of comparison of O1_cal6.4b and O1_cal6.4d

| ANOVA summary |  |
| --- | --- |
| F | 1173 |
| P value | <0.0001 |
| P value summary | **** |
| Significant diff. among means (P < 0.05)? | Yes |
| R squared | 0.9958 |

**Table S22.** One-way ANOVA parameters from comparisons of Cal6.4b and Cal6.4d

| **ANOVA table** | **SS** | **DF** | **MS** | **F (DFn, DFd)** | **P value** |
| --- | --- | --- | --- | --- | --- |
| Treatment (between columns) | 39800 | 4 | 9950 | F (4, 20) = 1173 | P<0.0001 |
| Residual (within columns) | 169.7 | 20 | 8.485 |  |  |
| Total | 39970 | 24 |  |  |  |

**Table S23.** Dunnett´s test from comparisons of Cal6.4b and Cal6.4d

| Dunnett's multiple comparisons test | Below threshold? | Summary | Adjusted P Value |
| --- | --- | --- | --- |
| Saline vs. O1_cal6.4d (1 mg/kg i.p.) | No | ns | 0.5145 |
| Saline vs. Normal Paw | Yes | **** | <0.0001 |
| Saline vs. O1_cal6.4b (1 mg/kg i.p.) | Yes | **** | <0.0001 |
| Saline vs. ω-MVIIA (0.5 mg/kg i.p.) | Yes | **** | <0.0001 |

1. **Supplementary figures**

**Figure S1.** RMSD of models a) cal6.4b and b) cal6.4d.
